# Mercury concentration in muscle, bellyfat and liver from *Oreochromis niloticus* and *Lates niloticus* consumed in Lake Albert fishing communities in Uganda

**DOI:** 10.1080/23311932.2016.1214996

**Published:** 2016-07-22

**Authors:** Tamale Andrew, Ejobi Francis, Muyanja Charles, Irene Naigaga, Nakavuma Jessica, Ocaido Micheal, Kato Charles Drago, Sente Celsus

**Affiliations:** ^a^ College of Veterinary Medicine, Animal Resources, and Biosecurity, Makerere University, P.O. Box. 7062, Kampala, Uganda; ^b^ College of Agricultural and Environmental Sciences, Makerere University, P.O. Box. 7062, Kampala, Uganda; ^c^ Middle East Technical University, Turkey

**Keywords:** heavy metal, fish, third world, Uganda, surveillance, consumption guideline

## Abstract

Without surveillance studies on mercury (Hg) levels in predominant fish species and parts eaten in a fishing community, the FAO/WHO guidelines might be surpassed, hence health risk. A monitoring study in a developing country with 29 *Oreochromis niloticus* (Nile tilapia) and 34 *Lates niloticus* (Nile perch) from landing sites provided muscle, bellyfat and liver samples for Mercury detection using Inductive Couple Plasma-optical emission spectroscopy. The study shows that fish eaten in the fishing community are small with fewer risks from mercury. Tilapia accumulated more mercury in muscle and liver than Nile perch. Fish consumed has mercury levels higher than FAO/WHO guidelines, and the bellyfat of Nile perch bioaccumulated more mercury than Tilapia. Based on the above, it is clear that some fish species should not be eaten by the vulnerable groups due to levels of Hg found in the muscle and bellyfat. This research will serve as a base for future studies, sensitization campaigns and policy design on mercury uptake through fish in fishing communities of developing countries.

## 1. Introduction

Mercury toxicity is one of most studied heavy metal contaminant of fish (Gimou et al., 2013). The issue of mercury toxicity through fish consumption has received considerable attention due to neurotoxicity, immune suppression and cognition issues in children less than five years (Engelberth et al., 2013; Paradis et al., 1997; Rheinberger & Hammitt, 2012).

Previous studies on mercury toxicity have reported the methods of detection, accumulation in different fish parts, species bioaccumulation differences, and the relationship to guideline values. The common methods of detection of Mercury levels include use of Mercury analyzer, ICP-OES (inductively coupled plasma-optical emission spectrometer, atomic absorption spectrophotometer, Instrument neutron activation analysis and graphite furnace atomic absorption spectrometry (Ababneh & Al-Momani, 2013; Bravo et al., 2010; Laar, Fianko, Akiti, Osae, & Brimah, 2011; Raissy & Ansari, 2014). Mercury accumulates differently in different fish species and fish parts. Some researchers believe that there is no difference in accumulation of mercury based on whether the fish is carnivorous or omnivorous, (Bidone, Castilhos, Santos, Souza, & Lacerda, 1997) while others have established a clear pattern of bioaccumulation of mercury in fish i.e. carnivorous fish have higher levels than the herbivorous and omnivorous fish a pointer towards the nutrition role in heavy metal toxicity (Zhu, Yan, Wang, & Pan, 2012). Looking now at the accumulation levels of mercury in the different parts of the fish i.e. muscles, liver, gills, kidney, brain and blood, there are differences in accumulation based on location, activities, level of pollution and species (Al Sayegh Petkovšek, Mazej Grudnik, & Pokorny, 2012; Mansilla-Rivera & Rodríguez-Sierra, 2011; Mieiro, Pacheco, Pereira, & Duarte, 2009; Sary & Mohammadi, 2011). However, to make meaning of the levels of the mercury found in fish parts need to compare with the international guidelines are available (Sary & Mohammadi, 2011).

The most common guidelines utilized to extrapolate the risk of exposure of mercury through fish consumption are: USA Environment Protection Agency, World Health Organization (WHO), Food and Agriculture Organization (FAO), and Food and Drug Administration (FDA) (Agusa et al., 2005; Bravo et al., 2010; Sary & Mohammadi, 2011; Sidhu, 2003; Weldemariam, 2012). Based on these guidelines, various messages about fish diets have been sent to the community.

According to Wheatley & Wheatley (2000) revealed that there was no direct linkage between mere presence of mercury in fish with the severe consequences documented.

One of the main obstacles is no studies conducted on heavy metals in the fishing community with effect of health issues at developing country. Much uncertainty still exists about the mercury levels in fish consumed in the fishing communities, hence a possibility of consuming contaminated products without their knowledge (Campbell, Verburg, Dixon, & Hecky, 2008; Ozuni et al., 2011). This paper argues that without surveillance studies on mercury levels in predominant fish species and parts eaten in a fishing community, the FAO/WHO recommended guideline levels might be surpassed hence possible health risk**.**

## 2. Materials and methods

### 2.1. Study area

**Figure 1.**
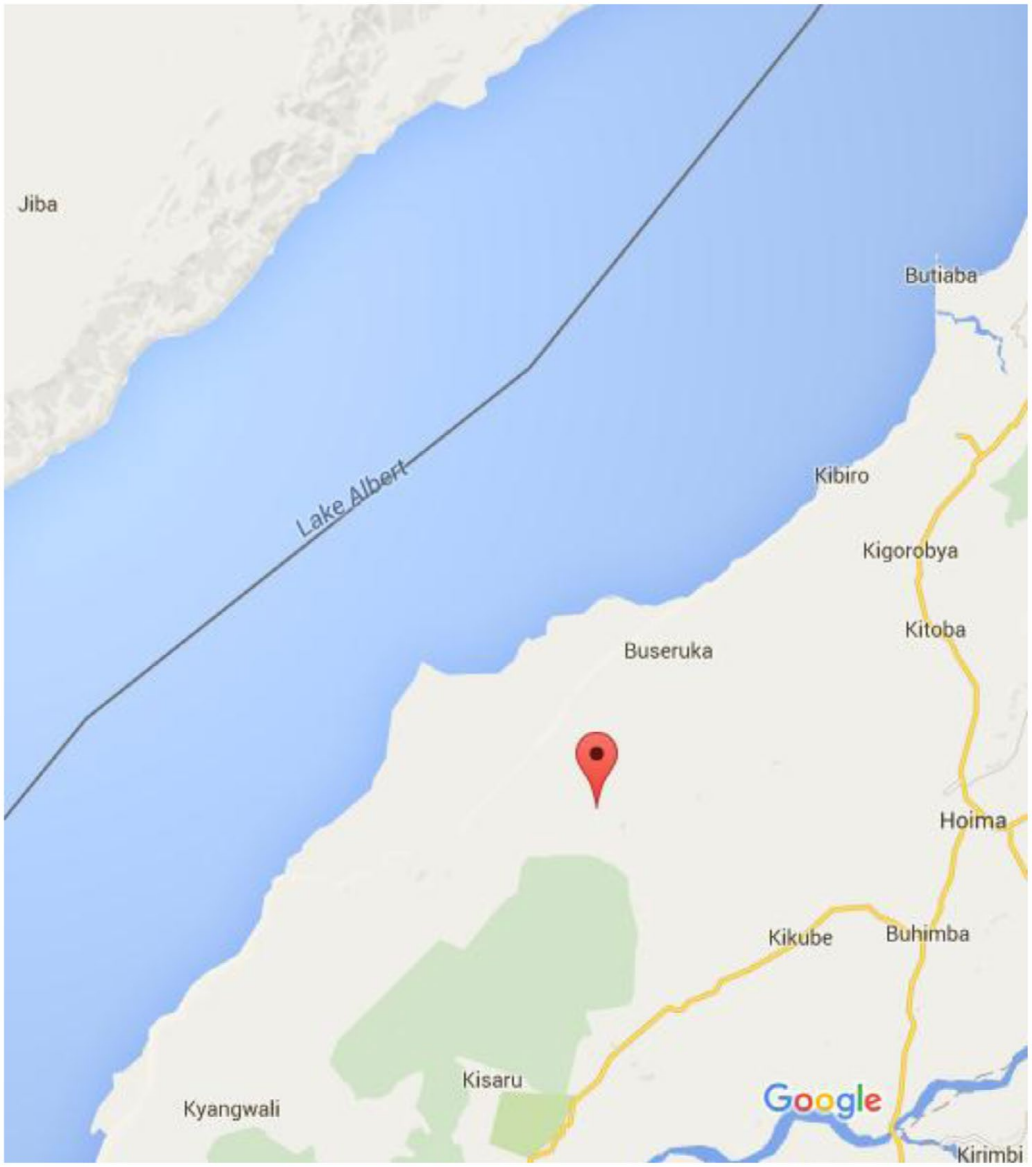
Landing sites in Hoima district Uganda.

Source: Adapted from Google Maps ... (Google (Cartographer), 2016).

This study was carried out in the Lake Albert fishing communities in Uganda. Lake Albert (Figure 1) is located in western Uganda, along 1.52 N; 30.86 E and is 10 km long, and occupies the most north part of the rift valley (Karp, Scholz, & McGlue, 2012).

The study site was Hoima district which is composed of 12 Sub Counties, four (Kyangwali, Kabwoya, Buseruka and Kigolobya) of which have access to the lake. The corresponding landing sites are Buhuka, Nkondo, Kaiso Tonya and Kibiro respectively. This site was selected because of the ongoing oil activities in the area and the absence of any study on mercury in the area.

### 2.2. Study design

This was a cross-sectional study. Sample collection was carried out in the months of March to May 2015 and then the samples taken to Government Analytical Lab in Wandegeya Kampala for mercury analysis.

### 2.3. Samples collection

A total of 63 fish species (*Oreochromis niloticus* (*n* = 29) and *Lates niloticus* (*n* = 34)) were collected from the landing site (Campbell, Dixon, & Hecky, 2003). The total length, standard length, and weight were measured for each fish using a standard board and weighing scale (Campbell, Balirwa, Dixon, & Hecky, 2004). Thereafter the fish were dissected to get about 30 g from the muscle below the dorsal fin. The muscle, bellyfat and liver were extracted into sterile glass bottles, sealed and labelled and put on ice in cool boxes maintaining the temperature range 0–4°C at the laboratory (Campbell et al., 2003; Kidd, Bootsma, Hesslein, Lyle Lockhart, & Hecky, 2003).

### 2.4. Analysis of samples

Fish samples were weighed before and after being cut into smaller pieces and samples were homoginized using a blender for homogenization. Five grams of the sample were put into a 125 ml Erlenmeyer flask (Garg, India), added 10 ml of concentrated nitric acid and warmed on a hot plate (Garg, India) until the tissue solubilized and samples were digested according to the (Campbell et al., 2003) protocol. The sample was topped up using deionized water to the mark for Inductive Couple Plasma- optical emission spectroscopy analysis. The ICP-OES was executed as discussed by (Indrajit Sen & Shrivaastava, 2011). The mercury levels in fish parts were measured in mg/kg wetweights (Campbell et al., 2003).

### 2.5. Instrumentation

Inductively Coupled Plasma-Optical Emission Spectroscopy (ICP-OES) equipped with argon saturation assembly, CCD detector and 21 CFR 11 were used (Indrajit Sen & Shrivaastava, 2011).

### 2.6. Method quality assurance

**Table:** 

**Matrix**	**Analyte**	**LOD**	**LOQ**	**Recovery range%**	**RSD**_**r**_**% (*n* = 63)**	**Accreditation Yes/No**
Fish muscle	Hg	ppb	ppb	94	3.3	Yes
Fish belly	Hg	ppb	ppb	94	2.7	Yes
Fish liver	Hg	ppb	ppb	96	5.6	Yes

### 2.7. Data analysis

Data on fish weight, total length, standard Length, fish species, muscle, bellyfat and liver amounts of mercury were checked for validity and reliability and analyzed (Table 1). Using Graph pad prism six The Linear model analysis was executed (Table 3) to yield a prediction model of mercury in the different fish parts. One-sample Wilcoxon tests were executed to determine the hypothesis in relation to FAO/WHO guideline values (*p* < 0.05) (Table 4).

**Table 1.** Fish parameters

Attributes	Tilapia	Nile perch
Sample size (*n*)	29	34
Median weight (mg)	790	226.5
Median total length (mm)	366	293.5
Median standard length (mm)	297.3	241.5

## 3. Results

### 3.1. Baseline attributes of the fish studied

The fish variables studied are species, length (total and standard) and Weight of the fish. These are key in providing information about the abundance of fish in the area. The Tilapia median weights (mg) were bigger than the Nile perch and upon using Mann Whitney test for comparison of the median weights, there was a significant difference in the weights amongst species (*p* < 0.0001). The median values of the weight, total length and standard length are shown in Table 1.

### 3.2. Level of mercury in the different parts of the fish

Mercury levels found in the fish analyzed were different for each part studied. The mercury levels in Tilapia muscle were 0.74 times than Nile perch. The liver accumulated mercury levels of Tilapia were 1.6 times than the Nile perch. The amount of mercury levels in Tilapia bellyfat were 0.8 times than the Nile perch. Kruskal wallis test was done for comparison of different fish parts and two medians from independent fish species populations showed no significant difference (Table 2).

**Table 2.** Level of mercury in fish species and parts studied in Lake Albert

Attributes	Tilapia	Nile perch	χ^2^	*p*-value
Median mercury muscle concentration (mg/g) & CI	0.0179 (0.014, 0.068)	0.024 (0.018, 0.033)	0.385	0.534
Median mercury liver concentration (mg/g) & CI	0.051 (0.019, 0.080)	0.031 (0.019, 0.044)	0.972	0.323
Median mercury bellyfat concentration (mg/g) & CI	0.016 (0.009, 0.036)	0.020 (0.017, 0.041)	2.449	0.117

### 3.3. Models of mercury in Lake Albert fish

Linear models were done for prediction of mercury in the different fish parts, species, the weight of the fish, the total length of the fish and standard length of the fish. The models revealed that the amount of mercury in the bellyfat could not be predicted by the variables above. Apart from the standard length, the other variables played a role in predicting the amount of mercury in both the liver and muscle. The amount of the variation that can be explained by the linear models is shown by the *R* ^2^ values shown in Table 3.

**Table 3.** Linear model predictors of mercury in the different fish parts

Attribute	Significant variable	*β*	SE	95% CI	Multiple *R*^2^
Bellyfat	None				0.09956
Liver	Intercept	1.348	0.340	(0.668, 2.027)	0.2215
	Species [Tilapia]	−0.261	0.120	(−0.502, −0.020)	
	Total length (mm)	−0.004	0.002	(−0.008, −0.0005)	
	Weight (gms)	0.0009	0.0004	(0.0002, 0.001)	
Muscle	Intercept	0.859	0.201	(0.456, 1.262)	0.228
	Species [Tilapia]	−0.175	0.071	(−0.317, −0.032)	
	Total length (mm)	−0.004	0.001	(−0.008, −0.0005)	
	Weight (gms)	0.0007	0.0002	(0.0002, 0.001)	

### 3.4. Relationship between selected international guideline values and levels of mercury in fish in Lake Albert

The values of mercury in the different fish parts was compared to set guideline values of FAO/WHO of 0.05 mg/kg. According to the median values obtained, no average was above the FAO/WHO guideline value. However, to establish whether there was a significant difference between the average amount and FAO/WHO guideline value using the Wilcoxon signed rank test, revealed significant difference for the Nile perch muscle and Tilapia bellyfat and Nile perch bellyfat was almost significant as shown in Table 4.

**Table 4.** Comparisons of mercury amounts in fish with different guideline values

Fish parts	Median amount of mercury (mg/kg) ± CI	^1^WHO guidelines on mercury amounts in solids (mg/kg)	^3^Australian consumption guidelines (mg/kg)	^2^European commission regulation (maximum level of mercury in muscle of fish (mg/kg))	Wilcoxon signed rank test for the parts of the fish against FAO/WHO standards (*p* < 0.05)
Tilapia muscle	0.018 (0.014, 0.068)	0.05	0.002	1.0	0.804
Tilapia bellyfat	0.016 (0.018, 0.036)	0.05	0.002	1.0	< 0.0001^*^
Nile perch muscle	0.024 (0.018, 0.033)	0.05	0.002	1.0	0.005*
Nile perch bellyfat	0.020 (0.017, 0.0417)	0.05	0.002	1.0	0.083

^1^ Joint FAO/WHO food standards programme codex committee on contaminants in foods.

^2^ Commission regulation (Ec) No 78/2005.

^3^ Food standards Australia New Zealand.

^*^ Refers to tissue samples which are significantly different from FAO/WHO guidelines.

However, some mercury values for the different fish parts were higher than the guideline values. All parts of the fish studied had values higher than the FAO/WHO recommended guidelines a pointer towards possible toxicity of fish. Using the FAO/WHO guideline of 0.05 mg/kg body weight, tilapia muscle was more contaminated than Nile perch i.e. ratio of 2:1.While the bellyfat of the Nile perch, was more contaminated than tilapia i.e. ratio of 7:1. The livers had almost similar contamination levels i.e. Tilapia was contaminated 10:8 times than Nile perch as displayed in Table 5.

**Table 5.** Ranges of mercury in different parts of fish

Mercury levels (mg/kg)	Muscle Nile perch	Muscle tilapia	Bellyfat Nile perch	Bellyfat tilapia	Liver Nile perch	Liver tilapia
≤0.01	13	15	17	14	11	9
0.011–0.05	15	5	8	10	13	10
0.051–1.0	4	9	7	1	8	10
>1	1	0	0	0	1	0

## 4. Discussion

The Lake Albert fishing community in Hoima district Uganda in 2010 experienced an outcry following an embargo on the purchase of foodstuffs from the local communities by the persons working in the oil companies. This embargo was perceived to be as a result of contamination of the foods include fish produced in the region. This embargo, presence of activities in the area i.e. oil exploration and charcoal burning, and the alleged perceived loss of taste of the fish from Lake Albert led to a surveillance research on the amount of mercury found in the predominant fish eaten in fishing community of a developing country (Hindrum, 2011).

The study found that the threat of mercury accumulation was not limited to the species as presumed by some researchers. This is in agreement with Bagumire et al. (2008) who looked at mercury in Catfish and Nile tilapia from farmed systems in Uganda, which had different feeding habits i.e. the Nile perch was piscivorous whereas the tilapia was omnivorous. Contrary to the expectations, the only fish factor significantly different between the species was the weights of the fish (*p* < 0.05). This finding revealed that the fishing community is not at risk of mercury exposure due to the sizes of the fish consumed. These finding are consistent with Burger et al. (2006).

The most obvious finding to emerge from the analysis is that mercury amounts were higher in Nile perch than in Tilapia for the whole fish (Table 2). A possible explanation for this is that the two fish species have different feeding practices. Nile perch is expected to accumulate more mercury levels if the small sized fish it consumes have accumulated considerable levels (Bonsignore et al., 2013). An implication of this is the possibility that consumption of carnivorous fish could predispose one to high amounts of mercury.

One interesting finding is that regarding the fish parts. Tilapia had more mercury in the muscle and liver than Nile perch while the reverse was true for the bellyfat (Table 5). However, this difference was not statistical difference between the species (*p* > 0.05). These results match those observed in earlier studies by (Bidone et al., 1997; Raissy, 2013) who studied accumulation of mercury in fish muscle and found out no species difference and that the defining factor for accumulation of the freshwater species was the season of the year. This finding further supports the idea that the possible sources of mercury observed in the fishing community are mainly from anthropogenic sources than natural sources (Hindrum, 2011). The liver of tilapia accumulated more mercury amounts than that of the Nile perch. Long time exposure and survival may be the reason for the higher accumulation of mercury in Nile perch. However, the findings of the current study do not support the previous research by Olivero-Verbel, Johnson-Restrepo, Mendoza-Marin, Paz-Martinez, and Olivero-Verbel (2004) These findings also contrast with Campbell, Osano, Hecky and Dixon (2003) who reported that carnivorous fish accumulate more mercury levels that detritivore fish i.e. tilapia due to the diet differences.

In contrast to earlier findings, however, mercury in bellyfat was not predicted by the fish factors i.e. length and weight. This result might be explained by the fact that no known research had demonstrated the relationship between the mercury amounts in bellyfat of the species studied. Further research should be undertaken to establish the relationship between mercury levels and other fish consumed in the community. The weight of the fish positively predicted the amount of mercury in fish liver. Total length and species were negatively correlated to mercury levels in tilapia and muscle of the two fish species (Table 3). These results are in line with Zhu et al. (2012) who looked at china fish species and found that age in addition to weight and length predicted mercury levels in fish muscle. The present study raises the possibility that large sized fish possessed more Mercury in Nile perch muscle (Sackett, Cope, Rice, & Aday, 2013).

The mere presence of mercury in fish does not guarantee toxicity in humans (Johnston & Snow, 2007). Therefore, there is need to relate the study findings to selected guideline and maximum values worldwide (Sary & Mohammadi, 2011). This relationship, while preliminary, suggests the levels of risks associated with consumption of fish in the community. One interesting finding is that, over 75% of the fish samples studied were above 0.002 mg/kg limit for mercury reporting in fish as per the Australian guidelines. It could be suggested that the community is already exposed to mercury risks and that this study should have been done way earlier to avoid the complications associated with mercury toxicity (Laar et al., 2011).

The mercury levels of 15, 31, 22 and 4% of Nile perch muscle, Tilapia muscle, Nile perch Bellyfat and Tilapia Bellyfat exceeded the guideline limits for FAO/WHO respectively (Table 5). However, the findings of the current study do not support the previous research by Laar et al. (2011) Therefore, constant monitoring for these metals was recommended due to the bioaccumulation in fish muscle. The study findings also contrast with Machiwa (2005) findings and recommendation that Nile perch is suitable for the vulnerable groups like pregnant women and children in the fishing community. This inconsistency may be due to the size and age of the fish that was used in the study (Bidone et al., 1997). Older Nile perch are expected to accumulate more amounts of mercury in the Bellyfat and muscle (Sackett et al., 2013). These findings have important implication for the vulnerable group i.e. HIV, pregnant women and children in the fishing community (Klein, 2005). In addition, the Bellyfat mercury amount of tilapia was higher than the FAO/WHO guidelines (Table 4). However, caution must be applied when utilizing the above findings. These might not be as conclusive as they appear since no work has previously been done on bellyfat part of the fish. The WHO/FAO guidelines were stipulated for muscle and may differ when it comes to bellyfat in fish. The study has been able to demonstrate that the levels of mercury in the fish liver were higher than the FAO/WHO guidelines a fact pointing towards fish health and breeding potential. These findings are contrasting from those of (Campbell et al., 2003; Kidd et al., 2003).

### 4.1. Limitation and strengths

The key strengths of this work are that it is the first heavy metal study in the fishing community of Uganda. The research also compared mercury guidelines for fish consumption advisory for vulnerable community in a developing country to that of FAO/WHO. The limitation could be that data was collected during the dry season since the Lake cannot be accessed by fishermen from December to March due to harsh weather.

## 5. Conclusions and recommendations

The purpose of this study was to evaluate the mercury levels in predominant fish consumed in a fishing community of a developing country in light of the FAO/WHO guidelines. The study has shown that the fish eaten in the fishing community are small sized fish with less risks from mercury. The research has also shown that the Nile perch accumulated more mercury in muscle and bellyfat than tilapia. This study has found that generally fish consumed has levels of mercury higher than the guideline values. One of the more significant findings to emerge from this study is that the bellyfat of tilapia and muscle of Nile perch accumulated more mercury than FAO/WHO guideline values.

The evidence from this study suggests that some fish species should not eaten by the vulnerable groups due to levels of Hg found in the muscle and belly fat. This finding has important implications for developing consumption guidelines in fishing communities of development countries.

This research will serve as a base for future studies, sensitization campaigns and policy design on mercury uptake through fish in fishing communities of developing countries.

### 5.1. Ethical consideration

This research was reviewed and approved by Institutional Review Board of College of Veterinary Medicine, Animal Resources and Biosecurity Makerere University under record number V:AB:REC/15/103. Final research approved by the Uganda National Council for Science and Technology under SIR 140.

## Public Interest Statement

The fear of consuming contaminated foods including fish is real to the Lake Albert fishing community. This fear is associated with the absence of information on the contaminants in predominant fish consumed, oil mining and illegal fishing activities. The study set out to establish the mercury amounts in the fish eaten and potential health risk. Purchases of two types of fish from landing sites markets were carried out. These fish are taken to a lab for mercury establishment. The community consumed small sized fish hence less mercury. However, large tilapia bellyfat and Nile perch muscle had higher mercury levels and should be avoided by young children and pregnant women. Therefore, knowledge of mercury levels in fish consumed is necessary to prevent health risks. This study sensitizes fishing communities in developing countries about fish risks.
